# Synaptic Activity and Bioenergy Homeostasis: Implications in Brain Trauma and Neurodegenerative Diseases

**DOI:** 10.3389/fneur.2013.00199

**Published:** 2013-12-11

**Authors:** Natasha Khatri, Heng-Ye Man

**Affiliations:** ^1^Department of Biology, Boston University, Boston, MA, USA; ^2^Department of Pharmacology and Experimental Therapeutics, Boston University School of Medicine, Boston, MA, USA

**Keywords:** glucose metabolism, glutamatergic neurotransmission, AMPK, mitochondria, Alzheimer disease, traumatic brain injury, stroke

## Abstract

Powered by glucose metabolism, the brain is the most energy-demanding organ in our body. Adequate ATP production and regulation of the metabolic processes are essential for the maintenance of synaptic transmission and neuronal function. Glutamatergic synaptic activity utilizes the largest portion of bioenergy for synaptic events including neurotransmitter synthesis, vesicle recycling, and most importantly, the postsynaptic activities leading to channel activation and rebalancing of ionic gradients. Bioenergy homeostasis is coupled with synaptic function via activities of the sodium pumps, glutamate transporters, glucose transport, and mitochondria translocation. Energy insufficiency is sensed by the AMP-activated protein kinase (AMPK), a master metabolic regulator that stimulates the catalytic process to enhance energy production. A decline in energy supply and a disruption in bioenergy homeostasis play a critical role in multiple neuropathological conditions including ischemia, stroke, and neurodegenerative diseases including Alzheimer’s disease and traumatic brain injuries.

## Introduction

The brain is the most energy-demanding organ in our body. It consumes 20% oxygen and 25% of total glucose supply, equivalent to approximately 20% of total ATP production (1–5). Given that the brain accounts for only 2% of our body weight, its energy consumption is impressive – 10 times that of other organs on average. The high cost in energy is not solely due to a large number of cells in the brain, with an estimated 100 billion neurons and many fold more glia, because organs with a comparable number of cells such as the liver have a much more modest energy bill (6). In contrast to peripheral tissues, neurons depend almost entirely on glucose for ATP production (1). Notably, the brain lacks cellular mechanisms to store energy or energy-generating sources such as glycogen or fat. Rather, energy must be produced continuously in order to maintain neuronal activity. Therefore, neurons are extremely sensitive to energy decline occurring during hypoxia, ischemia, stroke, and other forms of neurotrauma. Indeed, decreased glucose metabolism and mitochondrial energy production dysfunction have been associated with neurodegenerative diseases such as Alzheimer’s, Parkinson’s, and Huntington’s disease. Alzheimer’s and Huntington’s patients exhibit reduced glucose energy metabolism even at early stages of disease, possibly caused by reduced glucose uptake through transporters, mitochondrial dysfunction, or changes in mitochondrial motility. Traumatic brain injuries are becoming increasingly concerning in populations due to recent wars and the discovery of Chronic Traumatic Encephalopathy (CTE) in athletes. These conditions also cause rapid declines in neuronal glucose levels and associated long-term damaging effects, such as increased intracellular calcium, production of free radicals, and depolarization of the mitochondrial membrane. Recent studies have elucidated mechanisms in energy sensing and the role of synaptic events in energy metabolism and neuronal energy homeostasis, which shed light on our understanding of the pathogenesis of neurological diseases. In addition, proteins and pathways involved in neuronal energy metabolism are being investigated as therapeutic targets for neurodegenerative diseases and traumatic brain injuries.

## Glutamatergic Excitatory Synaptic Transmission is a Primary Energy-Consuming Event

Although glia outnumber neurons, the latter account for 85% of energy consumption (1). Among many neuronal cellular events, action potential-mediated neuronal communication is believed to be a major process of energy consumption. However, in contrast to a long-held belief, recent studies have revealed that the propagation of action potentials is highly energy efficient (7), consuming only 11% of brain ATP (8). Instead, energy cost mainly comes from synaptic activity, including transmitter release, but primarily postsynaptic receptor activation (9). In the brain, most of the synaptic activity is mediated by glutamate, thus, the excitatory glutamatergic system represents the single largest energy consumer, consuming 50% of ATP in the brain (4, 8, 10, 11). In addition to glutamate receptor channel activity, other glutamate-related events including glutamate synthesis, vesicle filling, release, uptake, and recycling, as well as receptor trafficking and signaling, are also energy consuming.

At the presynaptic terminals, glutamate is enriched in synaptic vesicles (SVs), powered indirectly by a proton pump on the vesicle membrane, at a concentration of 100 mM. During synaptic transmission, a single vesicle release can cause a rapid rise of glutamate in the synaptic cleft to concentrations as high as 1 mM (12). Under normal conditions, ambient glutamate in the extracellular environment is maintained by the constant activity of glutamate transporters at the plasma membrane of both neurons and glia (12, 13). Glial transporters often surround synapses to ensure an efficient uptake of released transmitter and prevent glutamate spillover.

There are three types of ligand-gated ionotropic glutamate receptors, including AMPA receptors (AMPARs), NMDA receptors (NMDARs), and kainate receptors (KRs) (14–16). AMPARs are sodium channels that are the major components responsible for synaptic transmission, whereas NMDARs play an essential role in the formation of synaptic plasticity, mainly via regulation of AMPAR trafficking and synaptic localization. More importantly, the high permeability of NMDARs to calcium enables the receptor to initiate a series of calcium-dependent signaling cascades, including those for energy-dependent protein modification and metabolic regulations (17). Of note, although NMDARs show high permeability to calcium and are often mistakenly considered a calcium channel, more than 80% of NMDA currents are actually carried by sodium (18). Since NMDA synaptic currents have a long-lasting time course compared to that of AMPARs, NMDARs contribute a large amount of sodium influx during synaptic activities.

A large amount of energy consumption results from the maintenance of ionic gradients via the sodium pump. Neuronal activity and synaptic transmission cause rises in intracellular sodium. Compared with the intracellular sodium concentration of about 10 mM at resting conditions, an action potential can increase spine sodium concentrations to 35–40 mM, and tetanus stimulation for the induction of long-term potentiation (100 Hz stimulation for 1 s) leads to sodium levels as high as 100 mM in the spine (19). Inhibition of the sodium pump activity abolishes glutamate-induced ATP reduction (20), indicating the sodium pump as the major cellular machinery attributing to glutamate-related energy spending. Membrane depolarization by glutamate stimulation induces firing of action potentials, which also leads to sodium influx via voltage-gated sodium channels. However, consistent with the notion that action potentials are energy efficient, blockage of sodium channels by tetrodotoxin (TTX) does not affect glutamate-induced ATP reduction, indicating that glutamate receptors are the primary source of intracellular sodium.

## Sensing of Cellular Energy by AMPK Signaling

When ATP is hydrolyzed to release energy to enable cellular processes, a rise in the AMP:ATP ratio is sensed by the bioenergy detector AMP-activated protein kinase (AMPK). Once activated, AMPK utilizes its serine/threonine kinase activity to increase the rate of cellular catabolism (glucose utilization, fatty acid oxidation, etc.) while simultaneously inhibiting anabolic processes (cell biosynthesis), resulting in a net increase in ATP production. AMPK is a heterotrimeric protein composed of α, β, and γ subunits in equal stoichiometry. The α subunit constitutes the catalytic domain, conferring kinase activity, while the γ subunit enables AMPK to monitor cellular energy status through two AMP/ATP binding domains, referred to as Bateman domains, that bind AMP or ATP in a mutually exclusive manner (21–23). An increase in the concentrations of AMP, an indicator of energy insufficiency, will facilitate AMP binding to the AMPK Bateman domains, leading to a change in molecular structure, and exposure of an activation loop in the α subunit. This conformational alteration allows AMPK to be phosphorylated at the α subunit Threonine 172 residue by upstream kinases, causing a 50–100-fold increase in the catalytic activity of AMPK (24). Conversely, a high concentration of intracellular ATP promotes ATP/Bateman domain binding and produces an antagonistic effect on AMPK activation. Given that neurons have a high degree of metabolic activity and energy demand, it is expected that AMPK plays a critical role in maintaining energy homeostasis within the brain.

AMPK can be phosphorylated by two upstream kinases including liver kinase B1 (LKB1) and the calmodulin-dependent protein kinase kinases, CaMKKα, and CaMKKβ (25–28). LKB1 was originally found as the tumor suppressor mutated in the genetically inherited susceptibility to human cancer, coined Peutz–Jeghers Syndrome (PJS) (29). In peripheral tissues, LKB1 has been shown to be necessary for AMPK phosphorylation and activation (30, 31). Despite both LKB1 and AMPK being ubiquitously expressed in mammalian cells, there is evidence to suggest that AMPK may be acted upon by different AMPKKs in a tissue-specific manner. For instance, LKB1 has been demonstrated to be the major upstream activator of AMPK in muscle and liver cells (32, 33), however a study utilizing LKB1 knockouts found that LKB1 deficient neurons had similar levels of phosphorylated AMPK as compared to wild-type cells under normal physiological conditions (34). In neurons, AMPK is more likely to be regulated by calcium-dependent signaling. In rat brain slices, intracellular increases in Ca^2+^ results in CaMKK-dependent AMPK phosphorylation. Importantly, membrane depolarization causes AMPK phosphorylation in the absence of an obvious change in cellular AMP:ATP ratio, indicating that AMPK can be regulated in a Ca^2+^-dependent, AMP-independent manner (35). Thus, glutamatergic synaptic activity can signal neurons for energy production via calcium-mediated AMPK activation.

## Coupling of Synaptic Activity and Energy Homeostasis

**Figure 1:**
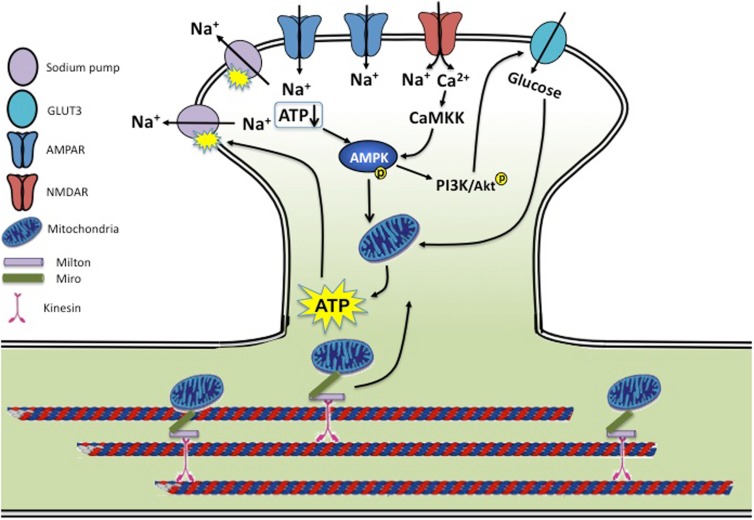
**Synaptic activity and energy homeostasis**. During synaptic transmission, activation of glutamate receptors allows influx of a large amount of sodium and calcium. Rises in intracellular sodium are rebalanced by the sodium pump powered by ATP consumption. Cellular energy status is sensed by AMPK via a reduced ATP/AMP ratio and CaMKK-dependent calcium signaling, leading to enhanced mitochondria activity and ATP biogenesis. AMPK activity also activates the PI3K/AKT pathway, leading to enhanced glucose uptake by stimulating glucose transporter membrane expression and transport efficiency. Mitochondria are trafficked on microtubules into metabolically demanding synapses by binding to Milton/Miro-mediated kinesin motor complex. In conditions of neurotrauma and neurodegenerative diseases, several aspects of this regulation may be disrupted. During hypoxia, ischemia, and stroke, insufficient ATP levels cause dysfunction of the sodium pump, leading to a loss in membrane potential and neuronal function. AD brains show reduced levels of GLUT3, and both AD and HD brains have a reduced rate of neuronal glucose metabolism. Mouse models of AD and PD show mitochondrial dysfunction along with reduced mitochondrial motility, preventing proper mitochondria delivery to the synapse and leading to decreased energy metabolism. Brains of traumatic injuries show reduced ATP levels and suppressed mitochondrial function.

In the brain, glutamate is the major neurotransmitter mediating most synaptic transmission. Multiple molecular events occurring during synaptic activation, including sodium pump activity, receptor trafficking, cytoskeletal rearrangements, signaling, and metabolic processes make synaptic activity an energetically costly process (8). Thus, coordinated cellular processes are necessary to convey synaptic signals to bioenergy metabolic activities (Figure 1).

### Co-ordination of sodium pump and glutamate receptor localization

The sodium gradient forms the foundation for synaptic transmission and neuronal excitation. Because of the frequent perturbation of ion homeostasis due to constant neuronal activity, the workload of the Na^+^/K^+^ ATPase (NKA) is so high that it consumes nearly half of the ATP in the brain. NKA is a heterodimer composed of two subunits: the catalytic α subunit that contains ATPase activity and the regulatory β subunit that is required for the enzymatic activity of NKA. At the single-neuron level, immunostainings have shown widespread localization of NKA in the soma and the dendrites (36, 37). During synaptic transmission, AMPAR-mediated currents are carried by sodium ions that flow into the cytosol of the neuron, typically within a microspace of the spine <1 μm^3^. In hippocampal neurons, one action potential can cause a several-fold increase in intraspinal sodium. The frequent and often large rises in intraspinal sodium must be exuded efficiently in order to maintain synapse electrophysiology, a task achieved via the activity of NKA. Therefore, there should exist cross-talk between AMPARs and the NKA to coordinate their functions. Indeed, we have shown that sodium pumps are enriched at the synapse and physically associate with AMPARs via interactions between the pump and receptor intracellular C-terminals. AMPAR surface localization and thus activity intensity are controlled to match the functional capacity of the pump. When sodium pump activity is decreased, AMPARs undergo a translocation from the plasma membrane to intracellular compartments via endocytosis, which are then directed to the proteasome for degradation. Presumably, the adjustment in surface glutamate receptor number can help prevent drastic toxicity caused by sodium and calcium accumulation due to sodium pump insufficiency. It remains unclear whether and how changes in glutamate receptor activity lead to corresponding regulation of NKA. However, changes in sodium pump levels correlating with glutamate receptor density have been documented. In the macaque retina, TTX treatment for 4 weeks caused a significant reduction in NMDARs; this reduction was paralleled by a lower level of NKA, suggesting that glutamate activity regulates NKA levels (38).

### Synaptic activation regulates glucose uptake

Glucose is the sole source for ATP production in neurons (1). Therefore, it is of physiological significance to have synaptic activity coupled with glucose uptake. Both neurons and glia are equipped with glucose utilization machinery, including glucose transporters and regulators, however higher glucose demands seem to be fulfilled with the assistance from glia. Glucose is first taken up by glia to be converted into lactate via glycolysis, which is then released and retaken by neurons where lactate is used for oxidative ATP genesis in mitochondria. Processes of astrocytes grow in the close proximity to neurons, often wrapping the synaptic cleft, as evidenced by a concentration of the astrocytic glucose transporter GLUT1 around synapses, where glutamate released during synaptic transmission is sensed by the glia and stimulate glial glucose uptake (39, 40).

In addition to the glia-coupled glucose delivery to neurons, synaptic activity can directly stimulate neuronal glucose uptake (41). However, exposure of neurons to glutamate results in a reduction in cellular ATP levels (20) and glucose uptake in neurons (42), indicating distinct signaling and cellular responses to synaptic vs. non-synaptic glutamate receptor activation.

AMPK is implicated in glutamate-induced glucose uptake. In neurons, AMPK signaling leads to activation of the PI3K/Akt pathway. We have shown that in cultured hippocampal neurons application of the AMPK activator AICAR causes a marked increase in phosphorylated Akt (43). This effect results directly from AMPK activation, as introduction of the AMPK antagonist successfully blocks AICAR-induced Akt phosphorylation. Furthermore, addition of a PI3K inhibitor also abolishes AICAR-induced Akt phosphorylation, indicating that the AMPK effect on Akt activation is mediated via PI3K (43) Interestingly, glutamate treatment activates AMPK, and pharmacological activation of AMPK leads to increased amounts of glucose transporters at the cell surface (44). We have recently found that in hippocampal neurons, AMPK activation causes higher levels of membrane GLUT3 and enhances glucose uptake (unpublished data). How AMPK activates PI3K remains unclear. Upon AICAR treatment, AMPK activation has been shown to phosphorylate IRS-1, the upstream component in the PI3K signaling pathway (45), suggesting IRS-1 as the intermediate factor linking AMPK to PI3K/Akt activation. Considering that glutamate-induced ATP reduction is a typical condition for AMPK activation (20, 46), the AMPK-PI3K-mediated enhancement in glucose uptake may function to prevent energy depletion and neuronal excitotoxicity. In addition, phosphorylated Akt may have a stimulatory effect on respiration by translocating to the mitochondria and increasing ATP synthase activity (47).

### Glutamate transporter and glutamate receptor activity in neuronal energy consumption

Glutamate is an extremely ample neurotransmitter, ranging to levels of 5–10 mmol/kg of brain tissue (48) and reaching millimolar concentrations within the synaptic cleft during synaptic transmission (49). However, glutamate levels are maintained in the micro- to nano-molar concentration in the extracellular milieu (50), many fold against its concentration gradient (12, 51–53). Unlike some neurotransmitters such as acetylcholine, which are efficiently removed by enzymatic digestion at the synaptic cleft, such disposal mechanism for glutamate does not exist. Instead, following release, glutamate is rapidly taken up by glia and neurons via membrane-distributed glutamate transporters (12, 54). By rapidly binding and transporting glutamate from the synaptic cleft, transporters limit the amount of glutamate receptor-permitted calcium influx and the subsequent excitotoxicity, a principal process involved in neuronal damage and neurodegeneration (55–57).

To date, five excitatory amino acid transports (EAAT1–5) have been identified in glia and neurons. The glial transporters EAAT1–2 are primarily localized to the plasma membrane of specialized domains in astrocytic processes (58, 59). The distribution of the neuronal transporters shows cell type specificity. EAAT3 is expressed in most neurons, including hippocampal and cortical neurons, whereas EAAT4 is mainly localized in cerebellar Purkinje cells and EAAT5 is restricted to the ribbon synapses of rod bipolar cells in the retina (60, 61). The majority of glutamate re-uptake is conducted by the glial transporters EAAT1 and EAAT2 (62, 63) which are expressed abundantly at the glial plasma membrane (59, 64) located in close proximity to synaptic release sites (65).

Glutamate transport by EAATs is powered indirectly by the sodium gradient across the membrane. During one complete cycle of glutamate transport, an EAAT brings one glutamate molecule against its concentration gradient, together with three Na^+^ ions and one H^+^ ion into the cell, meanwhile counter-transporting one K^+^ ion out of the cell, thereby resetting the transporter to the outward-facing conformation (66, 67). During stroke and brain trauma, a large amount of glutamate release is coupled with elevated activity of EAATs attempting to restore extracellular glutamate concentration. Despite EAAT activity being an ultimately energy consuming event, glutamate removal prevents overexcitation of glutamate receptors including AMPARs and NMDARs, which are ion channels with higher energy cost, and thus reduces net energy consumption. Indeed, inhibition of EAATs results in a decrease in ATP amount, which can be completely blocked by the glutamate receptor antagonists, indicating that local glutamate stimulation at synaptic sites causes ATP reductions similar to that caused by global glutamate application (20). Interestingly, glutamate uptake is powered mainly by glycolytic metabolism both in glia and neurons (68).

An additional layer of co-ordination exists between synaptic activity and glutamate receptor trafficking. In response to glutamate release and binding, glutamate receptors, especially the primary synaptic mediator AMPARs, undergo rapid translocation from the plasma membrane to the cytosolic domain via receptor internalization (69, 70). Elevated neuronal network activity or synaptic glutamate accumulation as a result of transporter suppression lead to AMPAR internalization (71). AMPAR trafficking has been extensively studied as a mechanism for synaptic plasticity and learning, but it may also play a role in energy homeostasis, especially in neurotraumatic conditions to prevent receptor overexcitation and rapid depletion of cellular energy store.

### Synaptic activity and mitochondria function and translocation

Mitochondria are responsible for generating and providing energy in the form of ATP in eukaryotic cells. In addition to converting glucose into ATP, mitochondria are involved in calcium signaling, apoptosis, and the metabolism of reactive oxygen species (ROS). With such high energy demands, neurons rely heavily on the proper functioning of mitochondria. The significance of this organelle in neurons has been shown by the implication of mitochondrial dysfunction in several neurodegenerative diseases (72). Mitochondria are also involved in other neurobiological processes including neural differentiation, neurite outgrowth, neurotransmitter release, and dendritic remodeling (73).

Because regions of highest energy consumption in the neuron are located at the synapses, mitochondrial transport and distribution are critical, since diffusion of ATP from the center of the neuron would be too slow and inefficient (74). Mitochondrial movement in dendrites is increased in areas with high levels of ATP and decreased in areas containing higher levels of ADP, suggesting that low levels of ATP signal the mitochondria to remain in the area so as to increase local energy supply (75). Dendrites contain a greater proportion of highly charged, more metabolically active mitochondria than axons to match energy demands of local activity. In accordance, axonal mitochondria are more mobile compared to those in the dendrites (76). This activity-dependent mitochondrial stopping results from NMDAR-gated calcium rises, which lead to a recruitment of mitochondria to the synapse (77). Mitochondria use the dynein and kinesin motor complexes to move in the retrograde and anterograde directions, respectively. Specifically, the core of this motor/adaptor complex is made up of kinesin-1, the protein Miro that is anchored to the outer surface of the mitochondria, and Milton, which links kinesin and Miro. A fine balance and regulation of the movements based on these complexes determine where mitochondria will be static or motile to provide adequate ATP for neuronal activity. Elevation of cytosolic Ca^2+^, which arises from activation of glutamate receptors in dendrites, stops both the anterograde and retrograde movement of mitochondria in neurons (77), which may be regulated by a Ca^2+^ binding site on Miro (78). How this regulation occurs remains unclear, although proposed mechanisms have included a conformational change in the complex triggered by Ca^2+^ (77), and direct binding of Ca^2+^ to kinesin, thereby preventing Miro from interacting with microtubules to allow mitochondrial movement (79).

Although less than synapses, axons themselves are also energy-demanding sites, as they are responsible for generating and conducting action potentials along the length of the neuron. In the peripheral nervous system, the nodes of Ranvier harbor the highest density of Na^+^ channels to sustain saltatory conduction (80). During action potentials, mitochondria are recruited to the nodal region and their mobility is reduced to provide more ATP (81). In addition, mitochondria motility seems to be crucial for axon growth and branching. A recent study shows that LKB1-NUAK1 signaling immobilize mitochondria in the axon where locally produced energy presumably supports formation of axon branches (82).

The regulation of mitochondrial function occurs both presynaptically and postsynaptically in the brain. In the presynaptic zone, the cycle of SVs in neuronal synapses involves steps regulated by cytosolic calcium concentrations and dependent on mitochondrial function. Upon the arrival of an action potential at the nerve terminal, voltage-gated Ca^2+^ channels open and allow an influx of calcium into the terminals. The elevated cytosolic calcium negatively affects mitochondria transport along microtubules, causing them to pause, and accumulate close to the active zones where SVs will fuse to the membrane (83). Synapses tend to have an accumulation of mitochondria that have high electrical potential across their inner membranes and are capable of enhanced ATP production (84).

Regulation of mitochondrial function in the postsynaptic region of the dendrite involves responses to glutamate to increase glucose uptake and ATP production. Synaptic activity increases surface expression of GLUT3 leading to an elevation of intracellular glucose (85). This effect is NMDAR-dependent and involves nNOS phosphorylated by Akt. As glutamate itself is utilized by mitochondria to produce ATP, the transport of glutamate into mitochondria is also regulated by activity. Interestingly, EAAT3 (EAAC1) has been shown to be expressed in neuronal and glial mitochondria where it participates in glutamate-stimulated ATP production (86).

## Energy Dysregulation in Ischemia and Stroke

Under normal conditions, high glutamate concentrations only occur at the synaptic cleft; ambient glutamate concentrations are maintained at very low levels (50). However, during traumatic brain injury (TBI) or stroke, massive glutamate release can lead to a marked increase in extracellular glutamate and hyperactivity of the overall glutamate system, causing additional acute and delayed neural pathology. Energy depletion plays a key role in glutamate-induced neurotoxicity (87–90). Glutamate stimulation causes more severe cell death when cellular energy homeostasis is impaired (88). A lack of sufficient ATP undermines a large number of energy-dependent cellular processes including kinase/enzymatic activity, proteasomal protein turnover, transmembrane biochemical gradients, and membrane potentials, all leading to a collapse of cellular functional integrity and deterioration of cell conditions. As the primary energy user consuming half of the ATP in the brain, sodium pump activity is highly sensitive to ATP levels. Under energy deficient conditions such as hypoxia, ischemia, and stroke, NKA dysfunction is often a major early pathological response (91, 92), which leads to a loss in membrane potential and neuronal function.

Ischemic stroke-induced energy depletion is sensed by the master metabolic regulator AMPK. AMPK activation has been observed in glutamate-treated neurons and a variety of ischemia/stroke models both *in vitro* (93) and *in vivo* (94). Because AMPK activation results in enhanced catalytic and suppressed anabolic metabolism, AMPK activity helps to relieve energy stress and is beneficial for neuronal conditions. Studies have shown that in cultured neurons AMPK activation reduces neuronal cell death caused by ischemia/hypoxia (93), whereas AMPK inhibition during energy stress stimulation leads to more severe damage (95). However, there are also studies showing deleterious effects of AMPK. *In vivo* ischemia model shows that blockade of AMPK by Compound C suppressed neural injury (96). Consistently, knockout of AMPK α2 results in a reduction of brain damage (97). Mechanisms for the detrimental effects of AMPK are not clear. Possibly, when cells are under conditions of metabolic stress, forced energy production pushes the metabolic machinery over its limits, causing a collapse of the system and irreversible structural and functional failure.

## Alterations of Bioenergy Metabolism in Neurodegenerative Diseases

Given that the brain is the major energy consumer in the body, and neurons rely heavily on ATP production for development and function, even a slight impairment in energy metabolism can have drastic effects on the brain. In line with this, mitochondria and bioenergy defects have long been proposed as the mechanism underlying chronic neuronal dysfunction and death, and an increasing amount of evidence has been accumulated in support of the hypothesis (Figure 1).

Alzheimer’s Disease (AD) is a neurodegenerative disease characterized by progressive memory loss and cognitive deficits. Its pathological hallmarks are neuronal loss, extracellular plaques consisting of Aβ aggregates and intracellular neurofibrillary tangles made up of hyperphosphorylated tau. Although the exact cause of neuronal death has not yet been determined, many studies suggest that dysfunction of energy metabolism may be responsible for neuronal deficits contributing to cell death. Indeed, AD patients exhibit reduced glucose energy metabolism, even at an early stage of disease. Positron emission tomography (PET) imaging with the 2-[18F]-fluorodeoxyglucose (FDG) tracer has long been used to track AD-related changes in the brain by estimating the cerebral metabolic rate of glucose (CMRglc). FDG-PET studies in AD show consistent and progressive CMRglc reductions. Compared to age-matched healthy controls, AD patients show metabolic reductions in the parieto-temporal and posterior cingulated cortices in early and late-onset AD (98, 99), and in the frontal areas in advanced disease (99–102). These changes in glucose metabolism could be caused by a reduction of glucose uptake through glucose transporters, mitochondrial dysfunction, or changes in mitochondrial movement.

The neuronal glucose transporter GLUT3 level is reduced in the AD brain (103). Full-length cAMP response element binding protein (CREB), which is reduced in AD brain along with an increase in the truncated form, regulates the expression of GLUT3. Calpain I proteolyses CREB at Gln28-Ala29 to generate a 41-kDa truncated CREB, which is less active in promoting GLUT3 expression, supported by the observation that activation of calpain I itself also reduces GLUT3 expression. It has been suggested that overactivation of calpain I by calcium overload proteolyses CREB, resulting in a reduction of GLUT3 expression, and consequently impairing glucose uptake and metabolism in AD brain (104). AMPK, as a sensor and regulator of cellular energy metabolism, has been shown to decrease with aging, and may contribute to decreased mitochondrial function in AD (105). A study using quercetin, a natural flavonoid and activator of AMPK, showed that activation of AMPK reduces oxidative stress, improves mitochondrial dysfunction and impaired glucose uptake in AD, and slows down Aβ accumulation (106).

Characterization of mitochondrial dynamics and function in three mouse models of familial AD (FAD) (APP, PS1, and APP/PS1) revealed mitochondrial dysfunction before the onset of memory phenotype and the formation of amyloid plaques (107). Movement of mitochondria in both anterograde and retrograde directions in FAD neurons was significantly inhibited compared to wild-type neurons. This reduced motility correlated with increased excitotoxic neuronal cell death by NMDA in all three FAD mouse models, consistent with the essential role for mitochondrial motility and positioning in proper calcium buffering (83). Additionally, similar effects were seen in mouse hippocampal neurons treated with the Aβ(23–35) peptide. Compared to the control neurons, which showed approximately 35% mobile mitochondria, motile mitochondria in the Aβ-treated neurons were significantly reduced to 20%, suggesting that the Aβ(25–35) peptide impairs axonal transport of mitochondria in AD neurons. This reduction in mitochondrial dynamics also correlated with, and was suggested to be causing, a reduction in synaptic proteins synaptophysin and MAP2. In the Tg2576 AD mouse model, where a significant decrease in mitochondrial movement was also seen (108), the mitochondria-targeted antioxidant SS31, which reduces intracellular free radicals (109), restored mitochondrial transport and synaptic viability, and decreased the percentage of defective mitochondria, implicating the important role of mitochondrial function in the disease. A recent report, however, found no consistent presynaptic bioenergetic deficiencies in three mouse models of AD pathogenesis (J20, Tg2576, and APP/PS1) (110). APP/PS1 cortical synaptosomes showed an increase in respiration associated with proton leak, but calcium handling and membrane potentials of synaptosomes were not consistently impaired. The disparities between these studies may be due to the mouse models used and the age of the animal when mitochondrial dysfunction was examined. In transgenic Drosophila expressing human tau, RNAi-mediated knockdown of Milton or Miro enhanced tau-induced neurodegeneration and increased tau phosphorylation at the AD-related site Ser262. Correlated with pathological conditions implicated in AD, a reduction in the number of axonal mitochondria was also observed, and knockdown of Miro alone was sufficient to induce late-onset neurodegeneration in the fly brain (111).

Parkinson’s disease (PD) is characterized pathologically by the selective degeneration of dopaminergic neurons in the substantia nigra pas compacta and the presence of Lewy bodies, intraneuronal aggregates comprised primarily of alpha-synuclein (α-syn). A mutation in α-syn, A53T, has been identified to cause familial Parkinson’s disease (112), and α-syn transgenic PD models display impaired mitochondrial function and decreased mitochondrial movement (113, 114). In addition, mutations in other Parkinson related proteins, such as PINK1, parkin, and DJ-1, are also believed to be involved in the regulation of mitochondrial function (115–117).

Huntington’s disease (HD) is an autosomal dominant neurodegenerative disease characterized by motor and cognitive impairment and caused by a trinucleotide repeat expansion encoding an elongated glutamine tract in the Huntingtin (htt) protein (118). Reduced energy metabolism has been well documented in HD patients. PET scan analysis of HD patients revealed diminished rates of cerebral glucose metabolism in parts of the cortex and throughout the striatum (119). Additionally, HD patient material was found to have significant reductions in the enzymatic activities of complexes II, III, and IV of the mitochondrial oxidative phosphorylation pathway in caudate and putamen (120, 121). BACHD mice of mutant Htt were found to have abnormal mitochondrial dynamics, supposedly due to the interaction of mutant Htt with the mitochondrial protein Drp1, resulting in defective anterograde movement (122). A major player implicated in mitochondrial dysfunction in Huntington’s, as well as Parkinson’s, is PPARγ co-activator-1α (PGC-1α). As a transcription co-activator, PGC-1α regulates the expression of various genes to promote mitochondrial biogenesis and oxidative phosphorylation. Impaired PGC-1α function is a likely contributor to HD pathology, as demonstrated by reduced PGC-1α target gene expression in HD transgenic mice (123). PGC-1α transcriptional activity is also repressed in a conditional knockout model of parkin (124), and activation of PGC-1α could rescue dopaminergic neuron loss induced by mutant α-syn (125). Consistently, PGC-1α has been suggested as a promising therapeutic target for HD and PD, either by boosting PGC-1α expression by viral delivery, or by modulating the upstream activators of PGC-1α activity, such as SIRT1 and AMPK (126).

## Implications of Energy Homeostasis in Traumatic Brain Injury

Traumatic brain injury (TBI) is a complex brain damage by an external force that causes brain penetrating or closed-head injuries. Recently, TBI has become an increasing concern in the population, as almost 179,000 service members sustained a TBI during the Iraq and Afghanistan wars (127). Additionally, repeated injury to the brain, especially concussions, can lead to CTE, a neurodegenerative disease that has been discovered in brain tissues of athletes who have sustained many close head and concussions injuries over time (128, 129). The complex mechanism by which TBI triggers pathological processes and long-term neurobehavioral abnormalities are still not well understood. Mechanistic investigation is critical to guide the identification of compounds to prevent acute neuronal damage and subsequent effects.

Traumatic brain injuries cause a vast array of primary structural damages that lead to secondary effects including cellular, inflammatory, neurochemical, and metabolic alterations. In the early phases after injury, changes such as metabolic impairment, reductions in cerebral blood flow, low ATP and energy stores, severe ionic shifts, and alterations in the permeability of the blood-brain barrier are seen. Thereafter, brain lactate production increases for the first few days, indicating a shift from aerobic to anaerobic metabolism to maintain ATP production, while glucose levels decline rapidly, as measured by microdialysis in affected patients (130). High levels of lactate in the brain during this period of ischemia may cause additional harmful effects; cerebral acidosis may exacerbate calcium-mediated damage to intracellular pathways and may interfere with ion-channel function (131). ATP levels are decreased following a TBI, along with reduced availability of the nicotinic coenzyme pool, which declines proportionally with the gravity of brain insult (132). The degree of oxidative metabolism depression also correlates with the depth of coma after severe TBI, as indicated by the Glasgow Coma Scale (GSC) (133). In mice, a single blast resulted in a 20% decrease in ATP levels in the cerebral cortex at 6 h after the blast, whereas triple blasts resulted in a similar decrease as early as 1 h (134). A significant, though less severe, decrease remained 24 h after the blast. Energy failure leads to degradation of molecules of key importance to membrane and cytoskeletal integrity. It also causes a disruption in ion homeostasis, especially calcium rises, and an increase in cytosolic acidity. The rise in free cytosolic Ca^2+^ is a result of failed calcium pump function, increased membrane permeability to calcium, and decreased sequestration of intracellular calcium. Elevated calcium levels and oxidative stress lead to the opening of the mitochondrial permeability transition pore (mPTP), which depolarizes the mitochondrial membrane and leads to organelle swelling and subsequent release of cytochrome *c*, leading to caspase-dependent cell death (135, 136). Specific inhibitors of the mPTP are currently under investigation as treatment immediately after TBI to prevent neuronal damage (137).

Mitochondrial dysfunction in TBI may be caused by several mechanisms in addition to the opening of mPTP. Nitric oxide (NO) is believed to cause respiratory chain inhibition in mitochondria after TBI (138), as it has the ability to interfere with energy metabolism by inhibiting the enzymatic activity of complex IV of the electron transport chain. An increase in NO production has been observed in closed-head trauma animal models (139), caused by the increase in the production of inducible NO synthase (iNOS) (140), as indicated by the rapid upregulation of iNOS mRNA at 4 h after injury. The inhibition of pyruvate dehydrogenase (PDH) has also been implicated in causing mitochondrial damage in TBI. PDH is tightly regulated by end-product inhibition and reversible phosphorylation, and a significant decrease in both PDH enzyme levels (141) and PDH phosphorylation (142) was found in rat TBI models. In addition, activation of poly(adenosine diphosphate [ADP]-ribose) polymerase-1 (PARP-1) could be responsible for impaired mitochondrial respiration. PARP-1 senses DNA damage after injury and becomes overactivated, depletes NAD^+^/NADH stores, and impairs the utilization of oxygen for ATP synthesis (143). In support of this mechanism, administration of NAD^−^ or the PARP inhibitor GP 6150 was found to be neuroprotective after TBI in rats (144, 145). Similar blockade of mitochondrial damage and metabolic disturbances in the early events occurring immediately after an injury are currently under investigation, which will be advanced following a better understanding of the molecular mechanisms underlying primary TBI impacts.

## Conclusion

Excitatory glutamatergic synaptic transmission is the major energy-consuming cellular process in the brain. Therefore, it is critical for neurons to couple synaptic activities with energetic metabolism, and to have adaptive mechanisms in response to metabolic stress and neuronal overexcitation. Dysfunctions in the regulatory system and bioenergy homeostasis can lead to defects in neural development and brain function, and contribute to the pathogenesis of neurodegenerative diseases and traumatic brain injuries. It will be important to further our understandings of how synaptic activity communicates with the metabolic and energetic machineries, including energy sensing, energetic signaling, bioenergy metabolism, and mitochondria dynamics. Age-dependent changes in bioenergy homeostasis, and epigenetic control of the energetic processes are also in need of further investigation.

## Conflict of Interest Statement

The authors declare that the research was conducted in the absence of any commercial or financial relationships that could be construed as a potential conflict of interest.

## References

[1] Attwell D Laughlin SB An energy budget for signaling in the grey matter of the brain. J Cereb Blood Flow Metab (2001) 21:1133–45 10.1097/00004647-200110000-00001 11598490

[2] Magistretti PJ Pellerin L Metabolic coupling during activation. A cellular view. Adv Exp Med Biol (1997) 413:161–6 10.1007/978-1-4899-0056-2_18 9238497

[3] Magistretti PJ Pellerin L Rothman DL Shulman RG Energy on demand. Science (1999) 283:496–7 10.1126/science.283.5401.496 9988650

[4] Raichle ME Gusnard DA Appraising the brain’s energy budget. Proc Natl Acad Sci U S A (2002) 99:10237–9 10.1073/pnas.172399499 12149485 PMC124895

[5] Rao J Oz G Seaquist ER Regulation of cerebral glucose metabolism. Minerva Endocrinol (2006) 31:149–58 16682938

[6] Elia M Organ and tissue contribution to metabolic rate. In: Kinney JM Tucker HN, editors. Energ Metabolism: Tissue Determinants and Cellular Corollaries. New York, NY: Raven Press (1992). p. 61–80

[7] Alle H Roth A Geiger JR Energy-efficient action potentials in hippocampal mossy fibers. Science (2009) 325:1405–8 10.1126/science.1174331 19745156

[8] Howarth C Gleeson P Attwell D Updated energy budgets for neural computation in the neocortex and cerebellum. J Cereb Blood Flow Metab (2012) 32:1222–32 10.1038/jcbfm.2012.35 22434069 PMC3390818

[9] Jolivet R Magistretti PJ Weber B Deciphering neuron-glia compartmentalization in cortical energy metabolism. Front Neuroenergetics (2009) 1:4 10.3389/neuro.14.004.2009 19636395 PMC2715922

[10] Shen J Petersen KF Behar KL Brown P Nixon TW Mason GF Determination of the rate of the glutamate/glutamine cycle in the human brain by in vivo 13C NMR. Proc Natl Acad Sci U S A (1999) 96:8235–40 10.1073/pnas.96.14.8235 10393978 PMC22218

[11] Sibson NR Dhankhar A Mason GF Rothman DL Behar KL Shulman RG Stoichiometric coupling of brain glucose metabolism and glutamatergic neuronal activity. Proc Natl Acad Sci U S A (1998) 95:316–21 10.1073/pnas.95.1.316 9419373 PMC18211

[12] Danbolt NC Glutamate uptake. Prog Neurobiol (2001) 65:1–105 10.1016/S0301-0082(00)00067-8 11369436

[13] Tzingounis AV Wadiche JI Glutamate transporters: confining runaway excitation by shaping synaptic transmission. Nat Rev Neurosci (2007) 8:935–47 10.1038/nrn2274 17987031

[14] Collingridge GL Isaac JT Wang YT Receptor trafficking and synaptic plasticity. Nat Rev Neurosci (2004) 5:952–62 10.1038/nrn1556 15550950

[15] Man HY Ju W Ahmadian G Wang YT Intracellular trafficking of AMPA receptors in synaptic plasticity. Cell Mol Life Sci (2000) 57:1526–34 10.1007/PL00000637 11092447 PMC11147035

[16] Newpher TM Ehlers MD Glutamate receptor dynamics in dendritic microdomains. Neuron (2008) 58:472–97 10.1016/j.neuron.2008.04.030 18498731 PMC2572138

[17] Kim MJ Dunah AW Wang YT Sheng M Differential roles of NR2A- and NR2B-containing NMDA receptors in Ras-ERK signaling and AMPA receptor trafficking. Neuron (2005) 46:745–60 10.1016/j.neuron.2005.04.031 15924861

[18] Skeberdis VA Chevaleyre V Lau CG Goldberg JH Pettit DL Suadicani SO Protein kinase A regulates calcium permeability of NMDA receptors. Nat Neurosci (2006) 9:501–10 10.1038/nn1664 16531999

[19] Rose CR Konnerth A NMDA receptor-mediated Na+ signals in spines and dendrites. J Neurosci (2001) 21:4207–14 11404406 10.1523/JNEUROSCI.21-12-04207.2001 PMC6762772

[20] Foo K Blumenthal L Man HY Regulation of neuronal bioenergy homeostasis by glutamate. Neurochem Int (2012) 61:389–96 10.1016/j.neuint.2012.06.003 22709672 PMC3430810

[21] Bateman A The structure of a domain common to archaebacteria and the homocystinuria disease protein. Trends Biochem Sci (1997) 22:12–3 10.1016/S0968-0004(96)30046-7 9020585

[22] Scott JW Hawley SA Green KA Anis M Stewart G Scullion GA CBS domains form energy-sensing modules whose binding of adenosine ligands is disrupted by disease mutations. J Clin Invest (2004) 113:274–84 10.1172/JCI19874 14722619 PMC311435

[23] Xiao B Heath R Saiu P Leiper FC Leone P Jing C Structural basis for AMP binding to mammalian AMP-activated protein kinase. Nature (2007) 449:496–500 10.1038/nature06161 17851531

[24] Hawley SA Davison M Woods A Davies SP Beri RK Carling D Characterization of the AMP-activated protein kinase kinase from rat liver and identification of threonine 172 as the major site at which it phosphorylates AMP-activated protein kinase. J Biol Chem (1996) 271:27879–87 10.1074/jbc.271.44.27879 8910387

[25] Baron SJ Li J Russell RR III Neumann D Miller EJ Tuerk R Dual mechanisms regulating AMPK kinase action in the ischemic heart. Circ Res (2005) 96:337–45 10.1161/01.RES.0000155723.53868.d2 15653571

[26] Clark SA Chen ZP Murphy KT Aughey RJ McKenna MJ Kemp BE Intensified exercise training does not alter AMPK signaling in human skeletal muscle. Am J Physiol Endocrinol Metab (2004) 286:E737–43 10.1152/ajpendo.00462.2003 14693511

[27] Woods A Dickerson K Heath R Hong SP Momcilovic M Johnstone SR Ca2+/calmodulin-dependent protein kinase kinase-beta acts upstream of AMP-activated protein kinase in mammalian cells. Cell Metab (2005) 2:21–33 10.1016/j.cmet.2005.06.005 16054096

[28] Woods A Vertommen D Neumann D Turk R Bayliss J Schlattner U Identification of phosphorylation sites in AMP-activated protein kinase (AMPK) for upstream AMPK kinases and study of their roles by site-directed mutagenesis. J Biol Chem (2003) 278:28434–42 10.1074/jbc.M303946200 12764152

[29] Avizienyte E Roth S Loukola A Hemminki A Lothe RA Stenwig AE Somatic mutations in LKB1 are rare in sporadic colorectal and testicular tumors. Cancer Res (1998) 58:2087–90 9605748

[30] Hawley SA Boudeau J Reid JL Mustard KJ Udd L Makela TP Complexes between the LKB1 tumor suppressor, STRAD alpha/beta and MO25 alpha/beta are upstream kinases in the AMP-activated protein kinase cascade. J Biol (2003) 2:28 10.1186/1475-4924-2-28 14511394 PMC333410

[31] Shaw RJ Kosmatka M Bardeesy N Hurley RL Witters LA DePinho RA The tumor suppressor LKB1 kinase directly activates AMP-activated kinase and regulates apoptosis in response to energy stress. Proc Natl Acad Sci U S A (2004) 101:3329–35 10.1073/pnas.0308061100 14985505 PMC373461

[32] Sakamoto K McCarthy A Smith D Green KA Grahame Hardie D Ashworth A Deficiency of LKB1 in skeletal muscle prevents AMPK activation and glucose uptake during contraction. EMBO J (2005) 24:1810–20 10.1038/sj.emboj.7600667 15889149 PMC1142598

[33] Shaw RJ Lamia KA Vasquez D Koo SH Bardeesy N Depinho RA The kinase LKB1 mediates glucose homeostasis in liver and therapeutic effects of metformin. Science (2005) 310:1642–6 10.1126/science.1120781 16308421 PMC3074427

[34] Barnes AP Lilley BN Pan YA Plummer LJ Powell AW Raines AN LKB1 and SAD kinases define a pathway required for the polarization of cortical neurons. Cell (2007) 129:549–63 10.1016/j.cell.2007.03.025 17482548

[35] Hawley SA Pan DA Mustard KJ Ross L Bain J Edelman AM Calmodulin-dependent protein kinase kinase-beta is an alternative upstream kinase for AMP-activated protein kinase. Cell Metab (2005) 2:9–19 10.1016/j.cmet.2005.05.009 16054095

[36] Anupama Adya HV Mallick BN Comparison of Na-K ATPase activity in rat brain synaptosome under various conditions. Neurochem Int (1998) 33:283–6 10.1016/S0197-0186(98)00043-6 9840218

[37] Brines ML Robbins RJ Cell-type specific expression of Na+, K(+)-ATPase catalytic subunits in cultured neurons and glia: evidence for polarized distribution in neurons. Brain Res (1993) 631:1–11 10.1016/0006-8993(93)91179-V 8298981

[38] Wong-Riley MT Huang Z Liebl W Nie F Xu H Zhang C Neurochemical organization of the macaque retina: effect of TTX on levels and gene expression of cytochrome oxidase and nitric oxide synthase and on the immunoreactivity of Na+ K+ ATPase and NMDA receptor subunit I. Vision Res (1998) 38:1455–77 10.1016/S0042-6989(98)00001-7 9667011

[39] Loaiza A Porras OH Barros LF Glutamate triggers rapid glucose transport stimulation in astrocytes as evidenced by real-time confocal microscopy. J Neurosci (2003) 23:7337–42 12917367 10.1523/JNEUROSCI.23-19-07337.2003 PMC6740433

[40] Morgello S Uson RR Schwartz EJ Haber RS The human blood-brain barrier glucose transporter (GLUT1) is a glucose transporter of gray matter astrocytes. Glia (1995) 14:43–54 10.1002/glia.440140107 7615345

[41] Bak LK Walls AB Schousboe A Ring A Sonnewald U Waagepetersen HS Neuronal glucose but not lactate utilization is positively correlated with NMDA-induced neurotransmission and fluctuations in cytosolic Ca2+ levels. J Neurochem (2009) 109(Suppl 1):87–93 10.1111/j.1471-4159.2009.05943.x 19393013

[42] Porras OH Loaiza A Barros LF Glutamate mediates acute glucose transport inhibition in hippocampal neurons. J Neurosci (2004) 24:9669–73 10.1523/JNEUROSCI.1882-04.2004 15509754 PMC6730152

[43] Amato S Liu X Zheng B Cantley L Rakic P Man HY AMP-activated protein kinase regulates neuronal polarization by interfering with PI 3-kinase localization. Science (2011) 332:247–51 10.1126/science.1201678 21436401 PMC3325765

[44] Weisova P Concannon CG Devocelle M Prehn JH Ward MW Regulation of glucose transporter 3 surface expression by the AMP-activated protein kinase mediates tolerance to glutamate excitation in neurons. J Neurosci (2009) 29:2997–3008 10.1523/JNEUROSCI.0354-09.2009 19261894 PMC6666202

[45] Jakobsen SN Hardie DG Morrice N Tornqvist HE 5’-AMP-activated protein kinase phosphorylates IRS-1 on Ser-789 in mouse C2C12 myotubes in response to 5-aminoimidazole-4-carboxamide riboside. J Biol Chem (2001) 276:46912–6 10.1074/jbc.C100483200 11598104

[46] Ioudina M Uemura E Greenlee HW Glucose insufficiency alters neuronal viability and increases susceptibility to glutamate toxicity. Brain Res (2004) 1004:188–92 10.1016/j.brainres.2003.12.046 15033434

[47] Li C Li Y He L Agarwal AR Zeng N Cadenas E PI3K/AKT signaling regulates bioenergetics in immortalized hepatocytes. Free Radic Biol Med (2013) 60:29–40 10.1016/j.freeradbiomed.2013.01.013 23376468 PMC3654039

[48] Butcher SP Hamberger A In vivo studies on the extracellular, and veratrine-releasable, pools of endogenous amino acids in the rat striatum: effects of corticostriatal deafferentation and kainic acid lesion. J Neurochem (1987) 48:713–21 10.1111/j.1471-4159.1987.tb05575.x 2879888

[49] Clements JD Lester RA Tong G Jahr CE Westbrook GL The time course of glutamate in the synaptic cleft. Science (1992) 258:1498–501 10.1126/science.1359647 1359647

[50] Herman MA Jahr CE Extracellular glutamate concentration in hippocampal slice. J Neurosci (2007) 27:9736–41 10.1523/JNEUROSCI.3009-07.2007 17804634 PMC2670936

[51] Kanner BI Schuldiner S Mechanism of transport and storage of neurotransmitters. CRC Crit Rev Biochem (1987) 22:1–38 10.3109/10409238709082546 2888595

[52] Kanner BI Sharon I Active transport of L-glutamate by membrane vesicles isolated from rat brain. Biochemistry (1978) 17:3949–53 10.1021/bi00600a011 708689

[53] Stern JR Eggleston LV Accumulation of glutamic acid in isolated brain tissue. Biochem J (1949) 44:410–8 PMC1274882 16748538

[54] Schousboe A Transport and metabolism of glutamate and GABA in neurons are glial cells. Int Rev Neurobiol (1981) 22:1–45 10.1016/S0074-7742(08)60289-5 6115823

[55] Arundine M Tymianski M Molecular mechanisms of calcium-dependent neurodegeneration in excitotoxicity. Cell Calcium (2003) 34:325–37 10.1016/S0143-4160(03)00141-6 12909079

[56] Choi DW Excitotoxic cell death. J Neurobiol (1992) 23:1261–76 10.1002/neu.480230915 1361523

[57] Man HY GluA2-lacking, calcium-permeable AMPA receptors – inducers of plasticity? Curr Opin Neurobiol (2011) 21:291–8 10.1016/j.conb.2011.01.001 21295464 PMC3092818

[58] Chaudhry FA Lehre KP van Lookeren Campagne M Ottersen OP Danbolt NC Storm-Mathisen J Glutamate transporters in glial plasma membranes: highly differentiated localizations revealed by quantitative ultrastructural immunocytochemistry. Neuron (1995) 15:711–20 10.1016/0896-6273(95)90158-2 7546749

[59] Rothstein JD Martin L Levey AI Dykes-Hoberg M Jin L Wu D Localization of neuronal and glial glutamate transporters. Neuron (1994) 13:713–25 10.1016/0896-6273(94)90038-8 7917301

[60] Hasegawa J Obara T Tanaka K Tachibana M High-density presynaptic transporters are required for glutamate removal from the first visual synapse. Neuron (2006) 50:63–74 10.1016/j.neuron.2006.02.022 16600856

[61] Wadiche JI Jahr CE Patterned expression of Purkinje cell glutamate transporters controls synaptic plasticity. Nat Neurosci (2005) 8:1329–34 10.1038/nn1539 16136036

[62] Bergles DE Jahr CE Glial contribution to glutamate uptake at Schaffer collateral-commissural synapses in the hippocampus. J Neurosci (1998) 18:7709–16 9742141 10.1523/JNEUROSCI.18-19-07709.1998 PMC6792997

[63] Rothstein JD Dykes-Hoberg M Pardo CA Bristol LA Jin L Kuncl RW Knockout of glutamate transporters reveals a major role for astroglial transport in excitotoxicity and clearance of glutamate. Neuron (1996) 16:675–86 10.1016/S0896-6273(00)80086-0 8785064

[64] Lehre KP Danbolt NC The number of glutamate transporter subtype molecules at glutamatergic synapses: chemical and stereological quantification in young adult rat brain. J Neurosci (1998) 18:8751–7 9786982 10.1523/JNEUROSCI.18-21-08751.1998 PMC6793562

[65] Ventura R Harris KM Three-dimensional relationships between hippocampal synapses and astrocytes. J Neurosci (1999) 19:6897–906 10436047 10.1523/JNEUROSCI.19-16-06897.1999 PMC6782870

[66] Levy LM Warr O Attwell D Stoichiometry of the glial glutamate transporter GLT-1 expressed inducibly in a Chinese hamster ovary cell line selected for low endogenous Na+-dependent glutamate uptake. J Neurosci (1998) 18: 9620–8 9822723 10.1523/JNEUROSCI.18-23-09620.1998 PMC6793325

[67] Zerangue N Kavanaugh MP Flux coupling in a neuronal glutamate transporter. Nature (1996) 383:634–7 10.1038/383634a0 8857541

[68] Schousboe A Sickmann HM Bak LK Schousboe I Jajo FS Faek SA Neuron-glia interactions in glutamatergic neurotransmission: roles of oxidative and glycolytic adenosine triphosphate as energy source. J Neurosci Res (2011) 89:1926–34 10.1002/jnr.22746 21919035

[69] Carroll RC Beattie EC von Zastrow M Malenka RC Role of AMPA receptor endocytosis in synaptic plasticity. Nat Rev Neurosci (2001) 2:315–24 10.1038/35072500 11331915

[70] Lissin DV Gomperts SN Carroll RC Christine CW Kalman D Kitamura M Activity differentially regulates the surface expression of synaptic AMPA and NMDA glutamate receptors. Proc Natl Acad Sci U S A (1998) 95:7097–102 10.1073/pnas.95.12.7097 9618545 PMC22752

[71] Jarzylo LA Man HY Parasynaptic NMDA receptor signaling couples neuronal glutamate transporter function to AMPA receptor synaptic distribution and stability. J Neurosci (2012) 32:2552–63 10.1523/JNEUROSCI.3237-11.2012 22396428 PMC3567454

[72] Chen H Chan DC Mitochondrial dynamics – fusion, fission, movement, and mitophagy – in neurodegenerative diseases. Hum Mol Genet (2009) 18:R169–76 10.1093/hmg/ddp326 19808793 PMC2758711

[73] Cheng A Hou Y Mattson MP Mitochondria and neuroplasticity. ASN Neuro (2010) 2:e00045 10.1042/AN20100019 20957078 PMC2949087

[74] Kuiper JW Pluk H Oerlemans F van Leeuwen FN de Lange F Fransen J Creatine kinase-mediated ATP supply fuels actin-based events in phagocytosis. PLoS Biol (2008) 6:e51 10.1371/journal.pbio.0060051 18336068 PMC2265766

[75] MacAskill AF Kittler JT Control of mitochondrial transport and localization in neurons. Trends Cell Biol (2010) 20:102–12 10.1016/j.tcb.2009.11.002 20006503

[76] Overly CC Rieff HI Hollenbeck PJ Organelle motility and metabolism in axons vs dendrites of cultured hippocampal neurons. J Cell Sci (1996) 109(Pt 5):971–80 8743944 10.1242/jcs.109.5.971

[77] Macaskill AF Rinholm JE Twelvetrees AE Arancibia-Carcamo IL Muir J Fransson A Miro1 is a calcium sensor for glutamate receptor-dependent localization of mitochondria at synapses. Neuron (2009) 61:541–55 10.1016/j.neuron.2009.01.030 19249275 PMC2670979

[78] Fransson S Ruusala A Aspenstrom P The atypical Rho GTPases Miro-1 and Miro-2 have essential roles in mitochondrial trafficking. Biochem Biophys Res Commun (2006) 344:500–10 10.1016/j.bbrc.2006.03.163 16630562

[79] Wang X Schwarz TL The mechanism of Ca2+ -dependent regulation of kinesin-mediated mitochondrial motility. Cell (2009) 136:163–74 10.1016/j.cell.2008.11.046 19135897 PMC2768392

[80] Fabricius C Berthold CH Rydmark M Axoplasmic organelles at nodes of Ranvier. II. Occurrence and distribution in large myelinated spinal cord axons of the adult cat. J Neurocytol (1993) 22:941–54 10.1007/BF01218352 7507976

[81] Zhang CL Ho PL Kintner DB Sun D Chiu SY Activity-dependent regulation of mitochondrial motility by calcium and Na/K-ATPase at nodes of Ranvier of myelinated nerves. J Neurosci (2010) 30:3555–66 10.1523/JNEUROSCI.4551-09.2010 20219989 PMC3548432

[82] Courchet J Lewis TL Jr Lee S Courchet V Liou DY Aizawa S Terminal axon branching is regulated by the LKB1-NUAK1 kinase pathway via presynaptic mitochondrial capture. Cell (2013) 153:1510–25 10.1016/j.cell.2013.05.021 23791179 PMC3729210

[83] Yi M Weaver D Hajnoczky G Control of mitochondrial motility and distribution by the calcium signal: a homeostatic circuit. J Cell Biol (2004) 167:661–72 10.1083/jcb.200406038 15545319 PMC2172592

[84] Lee CW Peng HB Mitochondrial clustering at the vertebrate neuromuscular junction during presynaptic differentiation. J Neurobiol (2006) 66:522–36 10.1002/neu.20245 16555236

[85] Ferreira JM Burnett AL Rameau GA Activity-dependent regulation of surface glucose transporter-3. J Neurosci (2011) 31:1991–9 10.1523/JNEUROSCI.1850-09.2011 21307237 PMC3045034

[86] Magi S Lariccia V Castaldo P Arcangeli S Nasti AA Giordano A Physical and functional interaction of NCX1 and EAAC1 transporters leading to glutamate-enhanced ATP production in brain mitochondria. PLoS One (2012) 7:e34015 10.1371/journal.pone.0034015 22479505 PMC3316532

[87] Baltan S Murphy SP Danilov CA Bachleda A Morrison RS Histone deacetylase inhibitors preserve white matter structure and function during ischemia by conserving ATP and reducing excitotoxicity. J Neurosci (2011) 31:3990–9 10.1523/JNEUROSCI.5379-10.2011 21411642 PMC3061553

[88] Del Rio P Montiel T Chagoya V Massieu L Exacerbation of excitotoxic neuronal death induced during mitochondrial inhibition in vivo: relation to energy imbalance or ATP depletion? Neuroscience (2007) 146:1561–70 10.1016/j.neuroscience.2007.03.024 17490821

[89] Nicholls DG Budd SL Mitochondria and neuronal glutamate excitotoxicity. Biochim Biophys Acta (1998) 1366:97–112 10.1016/S0005-2728(98)00123-6 9714760

[90] Nicholls DG Johnson-Cadwell L Vesce S Jekabsons M Yadava N Bioenergetics of mitochondria in cultured neurons and their role in glutamate excitotoxicity. J Neurosci Res (2007) 85:3206–12 10.1002/jnr.21290 17455297

[91] Mahadik SP Bharucha VA Stadlin A Ortiz A Karpiak SE Loss and recovery of activities of alpha+ and alpha isozymes of (Na(+) + K+)-ATPase in cortical focal ischemia: GM1 ganglioside protects plasma membrane structure and function. J Neurosci Res (1992) 32:209–20 10.1002/jnr.490320210 1328661

[92] Mrsic-Pelcic J Pelcic G Vitezic D Antoncic I Filipovic T Simonic A Hyperbaric oxygen treatment: the influence on the hippocampal superoxide dismutase and Na+,K+-ATPase activities in global cerebral ischemia-exposed rats. Neurochem Int (2004) 44:585–94 10.1016/j.neuint.2003.10.004 15016473

[93] Culmsee C Monnig J Kemp BE Mattson MP AMP-activated protein kinase is highly expressed in neurons in the developing rat brain and promotes neuronal survival following glucose deprivation. J Mol Neurosci (2001) 17:45–58 10.1385/JMN:17:1:45 11665862

[94] Harada S Fujita-Hamabe W Tokuyama S The importance of regulation of blood glucose levels through activation of peripheral 5’-AMP-activated protein kinase on ischemic neuronal damage. Brain Res (2010) 1351:254–63 10.1016/j.brainres.2010.06.052 20599814

[95] Wang P Xu TY Guan YF Tian WW Viollet B Rui YC Nicotinamide phosphoribosyltransferase protects against ischemic stroke through SIRT1-dependent adenosine monophosphate-activated kinase pathway. Ann Neurol (2011) 69:360–74 10.1002/ana.22236 21246601

[96] Li J Zeng Z Viollet B Ronnett GV McCullough LD Neuroprotective effects of adenosine monophosphate-activated protein kinase inhibition and gene deletion in stroke. Stroke (2007) 38:2992–9 10.1161/STROKEAHA.107.490904 17901380 PMC2637379

[97] McCullough LD Zeng Z Li H Landree LE McFadden J Ronnett GV Pharmacological inhibition of AMP-activated protein kinase provides neuroprotection in stroke. J Biol Chem (2005) 280:20493–502 10.1074/jbc.M409985200 15772080

[98] Ishii K Sasaki H Kono AK Miyamoto N Fukuda T Mori E Comparison of gray matter and metabolic reduction in mild Alzheimer’s disease using FDG-PET and voxel-based morphometric MR studies. Eur J Nucl Med Mol Imaging (2005) 32:959–63 10.1007/s00259-004-1740-5 15800784

[99] Sakamoto S Ishii K Sasaki M Hosaka K Mori T Matsui M Differences in cerebral metabolic impairment between early and late onset types of Alzheimer’s disease. J Neurol Sci (2002) 200:27–32 10.1016/S0022-510X(02)00114-4 12127672

[100] Foster NL Chase TN Mansi L Brooks R Fedio P Patronas NJ Cortical abnormalities in Alzheimer’s disease. Ann Neurol (1984) 16:649–54 10.1002/ana.410160605 6335378

[101] Friedland RP Budinger TF Ganz E Yano Y Mathis CA Koss B Regional cerebral metabolic alterations in dementia of the Alzheimer type: positron emission tomography with [18F]fluorodeoxyglucose. J Comput Assist Tomogr (1983) 7:590–8 10.1097/00004728-198308000-00003 6602819

[102] Minoshima S Giordani B Berent S Frey KA Foster NL Kuhl DE Metabolic reduction in the posterior cingulate cortex in very early Alzheimer’s disease. Ann Neurol (1997) 42:85–94 10.1002/ana.410420114 9225689

[103] Simpson IA Chundu KR Davies-Hill T Honer WG Davies P Decreased concentrations of GLUT1 and GLUT3 glucose transporters in the brains of patients with Alzheimer’s disease. Ann Neurol (1994) 35:546–51 10.1002/ana.410350507 8179300

[104] Jin N Qian W Yin X Zhang L Iqbal K Grundke-Iqbal I CREB regulates the expression of neuronal glucose transporter 3: a possible mechanism related to impaired brain glucose uptake in Alzheimer’s disease. Nucleic Acids Res (2013) 41:3240–56 10.1093/nar/gks1227 23341039 PMC3597642

[105] Jornayvaz FR Shulman GI Regulation of mitochondrial biogenesis. Essays Biochem (2010) 47:69–84 10.1042/bse0470069 20533901 PMC3883043

[106] Lu J Wu DM Zheng YL Hu B Zhang ZF Shan Q Quercetin activates AMP-activated protein kinase by reducing PP2C expression protecting old mouse brain against high cholesterol-induced neurotoxicity. J Pathol (2010) 222:199–212 10.1002/path.2754 20690163

[107] Trushina E Nemutlu E Zhang S Christensen T Camp J Mesa J Defects in mitochondrial dynamics and metabolomic signatures of evolving energetic stress in mouse models of familial Alzheimer’s disease. PLoS One (2012) 7:e32737 10.1371/journal.pone.0032737 22393443 PMC3290628

[108] Calkins MJ Manczak M Mao P Shirendeb U Reddy PH Impaired mitochondrial biogenesis, defective axonal transport of mitochondria, abnormal mitochondrial dynamics and synaptic degeneration in a mouse model of Alzheimer’s disease. Hum Mol Genet (2011) 20:4515–29 10.1093/hmg/ddr381 21873260 PMC3209824

[109] Cho S Szeto HH Kim E Kim H Tolhurst AT Pinto JT A novel cell-permeable antioxidant peptide, SS31, attenuates ischemic brain injury by down-regulating CD36. J Biol Chem (2007) 282:4634–42 10.1074/jbc.M609388200 17178711

[110] Choi SW Gerencser AA Ng R Flynn JM Melov S Danielson SR No consistent bioenergetic defects in presynaptic nerve terminals isolated from mouse models of Alzheimer’s disease. J Neurosci (2012) 32:16775–84 10.1523/JNEUROSCI.2414-12.2012 23175831 PMC3736741

[111] Iijima-Ando K Sekiya M Maruko-Otake A Ohtake Y Suzuki E Lu B Loss of axonal mitochondria promotes tau-mediated neurodegeneration and Alzheimer’s disease-related tau phosphorylation cia PAR-1. PLoS Genet (2012) 8:e1002918 10.1371/journal.pgen.1002918 22952452 PMC3431335

[112] Polymeropoulos MH Lavedan C Leroy E Ide SE Dehejia A Dutra A Mutation in the alpha-synuclein gene identified in families with Parkinson’s disease. Science (1997) 276:2045–7 10.1126/science.276.5321.2045 9197268

[113] Martin LJ Pan Y Price AC Sterling W Copeland NG Jenkins NA Parkinson’s disease alpha-synuclein transgenic mice develop neuronal mitochondrial degeneration and cell death. J Neurosci (2006) 26:41–50 10.1523/JNEUROSCI.4308-05.2006 16399671 PMC6381830

[114] Xie W Chung KK Alpha-synuclein impairs normal dynamics of mitochondria in cell and animal models of Parkinson’s disease. J Neurochem (2012) 122:404–14 10.1111/j.1471-4159.2012.07769.x 22537068

[115] Bonifati V Rizzu P van Baren MJ Schaap O Breedveld GJ Krieger E Mutations in the DJ-1 gene associated with autosomal recessive early-onset parkinsonism. Science (2003) 299:256–9 10.1126/science.1077209 12446870

[116] Kitada T Asakawa S Hattori N Matsumine H Yamamura Y Minoshima S Mutations in the parkin gene cause autosomal recessive juvenile parkinsonism. Nature (1998) 392:605–8 10.1038/33416 9560156

[117] Valente EM Abou-Sleiman PM Caputo V Muqit MM Harvey K Gispert S Hereditary early-onset Parkinson’s disease caused by mutations in PINK1. Science (2004) 304:1158–60 10.1126/science.1096284 15087508

[118] Norremolle A Riess O Epplen JT Fenger K Hasholt L Sorensen SA Trinucleotide repeat elongation in the Huntingtin gene in Huntington disease patients from 71 Danish families. Hum Mol Genet (1993) 2:1475–6 10.1093/hmg/2.9.1475 8242074

[119] Stoessl AJ Martin WR Clark C Adam MJ Ammann W Beckman JH PET studies of cerebral glucose metabolism in idiopathic torticollis. Neurology (1986) 36:653–7 10.1212/WNL.36.5.653 3486380

[120] Browne SE Bowling AC MacGarvey U Baik MJ Berger SC Muqit MM Oxidative damage and metabolic dysfunction in Huntington’s disease: selective vulnerability of the basal ganglia. Ann Neurol (1997) 41:646–53 10.1002/ana.410410514 9153527

[121] Gu M Gash MT Mann VM Javoy-Agid F Cooper JM Schapira AH Mitochondrial defect in Huntington’s disease caudate nucleus. Ann Neurol (1996) 39:385–9 10.1002/ana.410390317 8602759

[122] Shirendeb UP Calkins MJ Manczak M Anekonda V Dufour B McBride JL Mutant Huntingtin’s interaction with mitochondrial protein Drp1 impairs mitochondrial biogenesis and causes defective axonal transport and synaptic degeneration in Huntington’s disease. Hum Mol Genet (2012) 21:406–20 10.1093/hmg/ddr475 21997870 PMC3276281

[123] Weydt P Soyal SM Gellera C Didonato S Weidinger C Oberkofler H The gene coding for PGC-1alpha modifies age at onset in Huntington’s disease. Mol Neurodegener (2009) 4:3 10.1186/1750-1326-4-3 19133136 PMC2630305

[124] Shin JH Ko HS Kang H Lee Y Lee YI Pletinkova O PARIS (ZNF746) repression of PGC-1alpha contributes to neurodegeneration in Parkinson’s disease. Cell (2011) 144:689–702 10.1016/j.cell.2011.02.010 21376232 PMC3063894

[125] Zheng B Liao Z Locascio JJ Lesniak KA Roderick SS Watt ML PGC-1alpha, a potential therapeutic target for early intervention in Parkinson’s disease. Sci Transl Med (2010) 2:52ra73 10.1126/scitranslmed.3001059 20926834 PMC3129986

[126] Tsunemi T La Spada AR PGC-1alpha at the intersection of bioenergetics regulation and neuron function: from Huntington’s disease to Parkinson’s disease and beyond. Prog Neurobiol (2012) 97:142–51 10.1016/j.pneurobio.2011.10.004 22100502 PMC3506171

[127] Warden D Military TBI during the Iraq and Afghanistan wars. J Head Trauma Rehabil (2006) 21:398–402 10.1097/00001199-200609000-00004 16983225

[128] McKee AC Cantu RC Nowinski CJ Hedley-Whyte ET Gavett BE Budson AE Chronic traumatic encephalopathy in athletes: progressive tauopathy after repetitive head injury. J Neuropathol Exp Neurol (2009) 68:709–35 10.1097/NEN.0b013e3181a9d503 19535999 PMC2945234

[129] Omalu BI DeKosky ST Minster RL Kamboh MI Hamilton RL Wecht CH Chronic traumatic encephalopathy in a National Football League player. Neurosurgery (2005) 57:128–34 10.1227/01.NEU.0000163407.92769.ED 15987548

[130] Goodman JC Valadka AB Gopinath SP Uzura M Robertson CS Extracellular lactate and glucose alterations in the brain after head injury measured by microdialysis. Crit Care Med (1999) 27:1965–73 10.1097/00003246-199909000-00041 10507626

[131] Siesjo BK Pathophysiology and treatment of focal cerebral ischemia. Part I: pathophysiology. J Neurosurg (1992) 77:169–84 10.3171/jns.1992.77.2.0169 1625004

[132] Tavazzi B Signoretti S Lazzarino G Amorini AM Delfini R Cimatti M Cerebral oxidative stress and depression of energy metabolism correlate with severity of diffuse brain injury in rats. Neurosurgery (2005) 56:582–9 10.1227/01.NEU.0000156715.04900.E6 15730584

[133] Obrist WD Langfitt TW Jaggi JL Cruz J Gennarelli TA Cerebral blood flow and metabolism in comatose patients with acute head injury. Relationship to intracranial hypertension. J Neurosurg (1984) 61:241–53 6737048 10.3171/jns.1984.61.2.0241

[134] Arun P Abu-Taleb R Oguntayo S Wang Y Valiyaveettil M Long J Acute mitochondrial dysfunction after blast exposure: potential role of mitochondrial glutamate oxaloacetate transaminase. J Neurotrauma (2013) 30(19):1645–51 10.1089/neu.2012.2834 23600763

[135] Singleton RH Zhu J Stone JR Povlishock JT Traumatically induced axotomy adjacent to the soma does not result in acute neuronal death. J Neurosci (2002) 22:791–802 11826109 10.1523/JNEUROSCI.22-03-00791.2002 PMC6758486

[136] Young W Role of calcium in central nervous system injuries. J Neurotrauma (1992) 9(Suppl 1):S9–25 1588635

[137] Leung AW Halestrap AP Recent progress in elucidating the molecular mechanism of the mitochondrial permeability transition pore. Biochim Biophys Acta (2008) 1777:946–52 10.1016/j.bbabio.2008.03.009 18407825

[138] Fink MP Bench-to-bedside review: cytopathic hypoxia. Crit Care (2002) 6:491–9 10.1186/cc1824 12493070 PMC153437

[139] Wada K Chatzipanteli K Kraydieh S Busto R Dietrich WD Inducible nitric oxide synthase expression after traumatic brain injury and neuroprotection with aminoguanidine treatment in rats. Neurosurgery (1998) 43:1427–36 10.1227/00006123-199812000-00096 9848857

[140] Petrov T Underwood BD Braun B Alousi SS Rafols JA Upregulation of iNOS expression and phosphorylation of eIF-2alpha are paralleled by suppression of protein synthesis in rat hypothalamus in a closed head trauma model. J Neurotrauma (2001) 18:799–812 10.1089/089771501316919166 11526986

[141] Sharma P Benford B Li ZZ Ling GS Role of pyruvate dehydrogenase complex in traumatic brain injury and Measurement of pyruvate dehydrogenase enzyme by dipstick test. J Emerg Trauma Shock (2009) 2:67–72 10.4103/0974-2700.50739 19561963 PMC2700588

[142] Xing G Ren M Watson WD O’Neill JT Verma A Traumatic brain injury-induced expression and phosphorylation of pyruvate dehydrogenase: a mechanism of dysregulated glucose metabolism. Neurosci Lett (2009) 454:38–42 10.1016/j.neulet.2009.01.047 19429050

[143] Barr TL Conley YP Poly(ADP-ribose) polymerase-1 and its clinical applications in brain injury. J Neurosci Nurs (2007) 39:278–84 10.1097/01376517-200710000-00004 17966294

[144] LaPlaca MC Zhang J Raghupathi R Li JH Smith F Bareyre FM Pharmacologic inhibition of poly(ADP-ribose) polymerase is neuroprotective following traumatic brain injury in rats. J Neurotrauma (2001) 18:369–76 10.1089/089771501750170912 11336438

[145] Won SJ Choi BY Yoo BH Sohn M Ying W Swanson RA Prevention of traumatic brain injury-induced neuron death by intranasal delivery of nicotinamide adenine dinucleotide. J Neurotrauma (2012) 29:1401–9 10.1089/neu.2011.2228 22352983 PMC5972775

